# Changes in the Quality and Microbial Communities of Precooked Seasoned Crayfish Tail Treated with Microwave and Biological Preservatives during Room Temperature Storage

**DOI:** 10.3390/foods13081256

**Published:** 2024-04-19

**Authors:** Banghong Wei, Yan Gao, Yao Zheng, Jinxiang Yu, Xuejun Fu, Hairong Bao, Quanyou Guo, Huogen Hu

**Affiliations:** 1 East China Sea Fisheries Research Institute, Chinese Academy of Fishery Sciences, Shanghai 200090, China; weibanghong@ecsf.ac.cn (B.W.); gy4269262021@126.com (Y.G.); zhengyao@ecsf.ac.cn (Y.Z.); 2 School of Marine Science and Fisheries, Jiangsu Ocean University, Lianyungang 222005, China; 3 Aquatic Conservation and Rescue Center of Jiangxi Province, Nanchang 330029, Chinafuxuejun721@163.com (X.F.); 4 College of Food Science and Technology, Shanghai Ocean University, Shanghai 201306, China; hrbao@shou.edu.cn

**Keywords:** precooked seasoned crayfish tail, microwave sterilization, biological preservatives, microbial composition, volatile compounds

## Abstract

The qualities of precooked foods can be significantly changed by the microorganisms produced during room temperature storage. This work assessed the effects of different antibacterial treatments (CK, without any treatment; microwave treatment, MS; microwave treatment and biological preservatives, MSBP) on the physicochemical properties and microbial communities of precooked crayfish tails during room temperature storage. Only the combination of microwave sterilization and biological preservatives significantly inhibited spoilage, as evidenced by the total viable count (4.15 log CFU/g) after 3 days of room temperature storage, which satisfied the transit time of most logistics companies in China. Changes in pH and TVB-N were also significantly inhibited in the MSBP group compared with those in the CK and MS groups. More than 30 new volatile compounds were produced in the CK groups during room temperature storage. However, in the MSBP groups, the volatile compounds were almost unchanged. The correlations between the microbial composition and volatile compounds suggested that specific bacterial species with metabolic activities related to amino acid, energy, cofactor, and vitamin metabolism, as well as xenobiotics biodegradation and metabolism, were responsible for the changes in volatile compounds. These bacteria included *Psychrobacter*, *Arthrobacter*, *Facklamia*, *Leucobacter*, *Corynebacterium*, *Erysipelothrix*, *Devosia*, *Dietzia*, and *Acidovorax*. Overall, our findings provide a foundation for the development of strategies to inhibit spoilage in precooked crayfish tails stored at room temperature.

## 1. Introduction

Crayfish (*Procambarus clarkii*) is highly valued in China for its tasty and nutritious qualities, and it is rich in protein and low in fat [1]. The crayfish breeding industry in China has witnessed remarkable growth, with a total output of approximately 2.89 million tons in 2022, accounting for 60% of the overall Crustacea output. However, the industry’s progress is hindered by the seasonal nature of crayfish production. To overcome this limitation, the production of off-season crayfish has gained momentum, allowing for the processing of frozen crayfish tails and precooked commodities [2]. These precooked products, known for their convenience and delightful taste, have captured the attention of consumers and enjoy a vast market. Nevertheless, challenges arise because of the high initial microbial load, protein content, and moisture levels in crayfish products, rendering them susceptible to spoilage microorganisms. This compromises product quality and poses potential health risks to consumers [3,4]. Therefore, it is crucial to effectively control microbial contamination in precooked crayfish tail products.

There are many methods used to control microbial contamination in precooked products. Thermal sterilization is widely used for eliminating microorganisms [5]. However, the quality of the products, such as texture, can be significantly affected during thermal sterilization [6,7]. Compared with traditional thermal sterilization, microwave processing is considered a more friendly method, which not only effectively reduces potential microorganisms but also helps retain the nutrients and quality of food [8,9]. Although the treatments above can be used to reduce the initial microorganism count in the product, residual microorganisms can still grow during storage and transportation, leading to spoilage. The growth of spoilage microorganisms during storage and transport can be effectively inhibited by controlling the storage temperature. Refrigerated conditions can be used to maintain the quality of raw rainbow trout fillets for up to 14 days [10]. Similarly, crayfish can be stored at 4 °C for extended periods of up to 24 h, compared with 6 h at 25 °C, as indicated by the levels of microorganisms and biogenic amines [11]. Freezing is another commonly used preservation method to extend the shelf life of food. Cooked crayfish can be stored at −20 °C for up to 6 months [12]. As it is convenient to maintain a low storage temperature in cooked crayfish products, most of these kinds of products are stored under freezing conditions to inhibit the growth of microorganisms. However, the formation of ice crystals during freezing and subsequent thawing has been found to impair food quality by damaging the cellular structure [13]. Additionally, the costs are significantly increased because of the maintenance of freezing conditions during transportation and storage. Therefore, it is important to develop optimal preservation techniques that minimize quality changes and costs during the storage and transportation of precooked crayfish tails.

To reduce the costs of precooked crayfish products during transportation, we are trying to develop an optimal processing technique that can control microbial contamination tails at room temperature. In recent years, natural preservatives derived from organisms have gained popularity in food preservation. One such example is the antimicrobial peptide nisin, which has shown great potential in preserving processed meat products because of its effective antibacterial activity and food safety characteristics [14]. Moreover, the antibacterial activity of nisin can be enhanced when combined with other agents, such as sodium lactate [15]. The techniques mentioned above provide valuable insights for controlling microorganisms in precooked crayfish tails. Our previous study analyzed the effects of microwave treatment time and different concentrations of sodium lactate on the microorganisms and qualities of precooked crayfish tails stored at room temperature. We found that 18 min of microwave treatment significantly reduced the flavor of the product [16]. Only 15 min of microwave sterilization combined with 4% sodium lactate and 0.5 g/kg nisin could effectively inhibit microbial growth and reproduction and the deterioration of crayfish tails during storage at room temperature [16]. Using these techniques, precooked crayfish tails could be stored at room temperature for 3 days according to the microorganisms, which satisfied the transit time of most logistics companies in China. Therefore, precooked crayfish tails can be transported at room temperature rather than cold-chain transportation, which reduces the cost of transportation.

The rapid proliferation of microorganisms promotes the spoilage of aquatic products during storage. The metabolites produced by these spoilage microorganisms result in a fishy odor and sensory aversion, which could be reflected by volatile compounds [17,18,19]. Although our previous study investigated effective treatments for inhibiting microbial growth and reproduction during the room temperature storage of precooked crayfish tails, changes in microbial communities were not analyzed. To further prolong the storage of precooked crayfish tails at room temperature, changes in the microbial communities in the products should be analyzed during storage. In this paper, we analyzed the effects of microwave and biological preservative treatments, which we previously used, on the changes in the quality (pH, total volatile basic nitrogen (TVB-N), and volatile compound profiles) and microbial communities (16S rRNA amplicon sequencing and bioinformatics analyses) of precooked crayfish tails stored at room temperature. Additionally, we investigated the spoilage microorganisms in precooked crayfish tails according to the relationship between the volatile compound profiles and microbial communities. Ultimately, the findings from this study can contribute to the development of optimal preservation techniques for precooked crayfish tails and facilitate their transportation at room temperature without compromising their quality.

## 2. Materials and Methods

### 2.1. Materials, Sample Preparation, and Storage

The crayfish tails were purchased from a commercial company. The crayfish tails were produced as follows: crayfish of similar weight were collected and boiled in boiling water for 2–3 min. Then, the boiled crayfish were cooled in cold water. The cephalothorax was removed, and the crayfish tails were frozen in liquid nitrogen. The tails were kept at −20 °C until they were processed. Precooked crayfish tails were prepared according to the methods of a commercial company and our previous study [16]. The seasoning solution is composed of oil and soup. The seasoned oil contained soybean oil, salt, sugar, scallion, celery, ginger, garlic, chili sauce, fine herbs, clove, fennel, aniseed, nutmeg, and other ingredients. The seasoned soup mainly contained water, sugar, salt, sodium D-isoascorbate, gourmet powder, ginger powder, glycine, and other additives. To prepare the crayfish tails, a mixture of 4 parts of tails, 2 parts of seasoned oil, and 3 parts of seasoned soup was created. This mixture was then placed in polyester polyethylene trays and sealed with polyvinylchloride film using a modified atmosphere packaging machine (FD-Z1, Fudi, China). The gas used in this study consisted of 80% CO_2_, 15% N_2_, and 5% O_2_. For the control group (CK), the packaged precooked crayfish tails were used as is. For the microwave sterilization group (MS), the packaged precooked crayfish tails were treated with a microwave sterilizer (YQ7G-05, Nanjin Yongqing Food New Technology Development Co., Ltd., Nanjin, China) using a power of 900 W. The temperature was set at 95 °C for 15 min. After microwave sterilization, the precooked crayfish tails were cooled in ice water. For the microwave sterilization combined with biological preservatives group (MSBP), 4% sodium lactate (*w*/*w*) and 0.05% nisin (*w*/*w*) were added to the seasoned soup. The microwave sterilization process was the same as that for the MS group. The packaged crayfish tails in trays were then stored at 25 °C for three days. Three trays from each group were collected at the beginning and every day during the storage period for further analysis and evaluation.

### 2.2. Determination of Total Viable Count

The total viable count (TVC) of the precooked crayfish tails was measured referring to the TVC detection method in the Chinese National Standard GB/T 4789.2 [20]. Twenty-five grams of crayfish tail (without shell) was homogenized with 225 mL of 0.85% sterile saline solution. Then, the homogenized samples were serially diluted, and 1 mL of the dilutions was spread onto the nutrient agar and incubated at 30 °C for 48–72 h. The colony counts were recorded. The TVC results were expressed as the logarithm base 10 colony forming units per gram (log CFU/g).

### 2.3. Chemical Analyses

After being stored at room temperature for 3 days, the crayfish tails were collected for further analysis. The method in Rajkumar’s paper was used to measure the pH of the crayfish tails [21]. The procedure involved homogenizing 5 g of precooked crayfish tails (shell removed) with 45 mL of ultrapure water for 1 min. The resulting homogenate was then filtered, and the pH of the filtrate was measured using a pH meter (pHS-3C, INESA Scientific Instrument Co., Ltd., Shanghai, China).

Total volatile basic nitrogen (TVB-N) was determined following the method described in the Chinese standard GB 5009.228-2016 using an automatic Kjeldahl apparatus (KDN-103F, Shanghai Xianjian Instrument Co., Ltd., Shanghai, China) [22]. A sample weighing 20 ± 0.001 g of crayfish tail (without shell) was homogenized in 100 mL of ultrapure water and allowed to sit at room temperature for 30 min. The homogenate was then filtered using filter paper. Then, 10 mL of the filtrate was mixed with 10 mL of water and 5 mL of MgO (10 g/L) and loaded onto an automatic nitrogen analyzer. The volatile basic nitrogen in the sample was absorbed by 10 mL of boric acid solution (20 g/L). After adding 5 drops of a mixed indicator, the resulting distillate was titrated with a standard hydrochloric acid solution (0.01 M) until a purple-red color change occurred. The TVB-N content (mg/100 g) was calculated using the following formula: TVB-N (mg/100 g) = 1.4 × (V − V_0_)/m, where m is the mass of the crayfish tail (without shell), the unit of which is grams. V and V_0_ are the volumes of the standard hydrochloric acid solution consumed in the sample and blank groups, respectively. The units of V and V_0_ are milliliters.

### 2.4. Volatile Compounds

The analysis of volatile organic compounds was conducted using a gas chromatography-ion migration spectrometry (GC-IMS) instrument from G.A.S., Dortmund, Germany, following the method described previously [19]. In this study, approximately 2 g of precooked crayfish tail (shell removed) was placed in a 20 mL headspace glass vial. The vial was then incubated at 40 °C for 15 min at a rotational speed of 500 rpm. A total of 500 μL of the extracted sample was injected into the GC-IMS system, with the syringe temperature set at 60 °C. To separate the volatile compounds, a gas chromatographic column (MXT-WAX, 30 m × 0.53 mm, 1 μm) was used. Nitrogen gas with a purity of ≥99.999% was used as the carrier gas. The column temperature was maintained at 60 °C throughout the 30 min run time. The flow rate of the carrier gas was set as follows: initially, 2 mL/min for 2 min, then increased to 10 mL/min at 10 min, further increased to 100 mL/min at 20 min, and maintained at 100 mL/min for the remaining 10 min. In the IMS ionization chamber equipped with a tritium ionization source, the volatile analytes underwent ionization. The drift gas, consisting of nitrogen with a purity of ≥99.999%, was maintained at a flow rate of 150 mL/min. The temperature of the drift tube was set at 45 °C.

Volatile compounds in the precooked crayfish tails were analyzed using GC-IMS and VOCal 1.0 software. The identification of these volatile compounds was based on the retention index (RI) and drift time, which were matched against the NIST and IMS databases. To quantify the volatile compounds, a fingerprinting analysis was conducted using the gallery plot plug-in. The intensity of the signal peak was utilized to represent the relative concentrations of the compounds. Additionally, the OPLS-DA method was employed to discriminate the differences in volatile compounds in different groups.

### 2.5. DNA Extraction and 16S rRNA Sequencing

After being stored at 25 °C for three days, the crayfish tails (without shells) were collected for microbial community analysis. Three samples were collected from each group for parallel analyses. Total DNA was extracted using the PowerFood Microbial DNA Isolation kit (Qiagen, Hilden, Germany) according to the manufacturer’s instructions and stored at −20 °C for future use. The quantity of DNA was measured using a Nanodrop spectrophotometer (Thermo Scientific, Waltham, MA, USA). Additionally, the quality of the DNA was verified through agarose gel electrophoresis.

Sequencing libraries were prepared using Q5^®^ High-Fidelity DNA Polymerase (NEB, Ipswich, MA, USA) to amplify the V3 and V4 regions with 338 F (ACTCCTACGGGAGGCAGCA) and 806 R (GGACTACHVGGGTWTCTAAT). The PCR conditions were as follows: initial denaturation at 98 °C for 2 min, followed by 25–30 cycles of denaturation at 98 °C for 15 s, annealing at 55 °C for 30 s, extension at 72 °C for 30 s, and final extension at 72 °C for 5 min. The reaction was then held at 10 °C. After purification and quantification, the PCR products were subjected to paired-end sequencing using the Illumina NovaSeq platform (San Diego, CA, USA).

### 2.6. Data Processing and Microbial Community Analysis

The raw data were acquired in FASTA format and subsequently analyzed using QIIME 2. Initially, the raw data underwent demultiplexing and primer trimming. Next, the sequences were subjected to thorough quality filtering, denoising, merging, and chimera removal using the DADA2 method [23]. De-duplication was performed, resulting in the generation of Amplicon Sequence Variants (ASVs) at 100% similarity. Classification of these ASVs was achieved by aligning representative sequences to the Greengenes database (Release 13.8) using the classify-sklearn function in QIIME2 [24]. To assess alpha diversity, Chao1 and Shannon indices were calculated. Beta diversity was evaluated using the Bray–Curtis dissimilarity index. The prediction of microbial functions was conducted using PICRUSt2, which compares microbial functions to the KEGG pathway database [25].

### 2.7. Statistical Analysis

All experiments were conducted with three biological replicates, and the results are presented as the mean ± standard deviation (SD). Statistical analysis was performed using a one-way analysis of variance, and the significance was determined using Duncan’s test in SPSS 22 (IBM, Armonk, NY, USA). Statistical significance was set at *p* < 0.05. Figures were constructed using GraphPad Prism 9 (GraphPad, La Jolla, CA, USA) and Origin Pro (OriginLab, Northampton, MA, USA).

## 3. Results and Discussion

### 3.1. Changes in the TVC during Room Temperature Storage of Precooked Crayfish Tails

During the storage of precooked crayfish tails at room temperature, the TVC was measured, and the results are presented in Table 1. The initial TVC in the CK group was 4.82 log CFU/g. As the storage time increased, the TVC drastically increased to 10.81 after 3 days. This indicated significant deterioration of the crayfish tail by the end of the third day. After applying microwave sterilization, the initial TVC was significantly lower than that in the CK group. However, even with microwave sterilization, the TVC rapidly increased and reached 6.62 after two days of room temperature storage. Compared with those in the CK and MS groups, the TVC in the MSBP group increased at a slower rate. The TVC in the MSBP group was only 4.15 log CFU/g after 3 days of room temperature storage, which was still below the threshold specified in the Chinese National Standard GB 10136 (2015) for read-to-eat aquatic products [26].

Because of the high initial TVC in the CK group, most manufacturers must transport precooked crayfish tails using cold-chain transportation, which significantly increases the cost. Microwave sterilization was used to deactivate microorganisms in the precooked crayfish tails. Although the microwave sterilization process was effective in reducing the initial microorganisms in the precooked crayfish tail, it failed to inhibit microbial growth during room temperature storage. Nisin, a substance known to affect microbial cell membranes by binding to the cell wall precursor Lipid II, causes cell death [27]. The antibacterial activity of nisin can be enhanced when combined with sodium lactate [15]. By adding nisin and sodium lactate, the microorganisms were effectively deactivated and inhibited in the precooked crayfish tails and could be stored at room temperature for 3 days. Thus, precooked crayfish tails can be transported without cold-chain transportation for 3 days, which satisfies the transit time requirements of most logistics companies in China.

### 3.2. Changes in pH and TVB-N Values during Room Temperature Storage of Precooked Crayfish Tails

**Figure 1:**
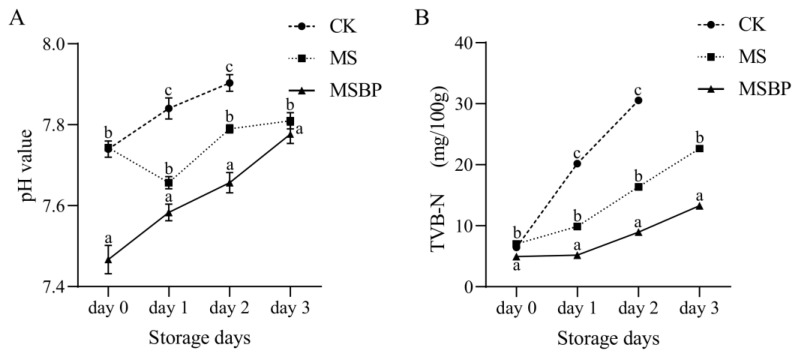
Changes in pH (**A**) and TVB-N (**B**) in precooked seasoned crayfish tails during storage at room temperature. Different letters in the figures indicate that there are significant differences between groups on the same day.

pH is an important indicator for assessing the freshness and quality of aquatic products. In this study, the pH increased with increasing storage time, except on day 1 in the MS group (Figure 1A). The reason for the decreased value in the MS group at the beginning of storage still needs to be further investigated. The increase in pH during storage can be attributed to microbial growth and the action of endogenous enzymes that lead to the breakdown of proteins in meat products, resulting in the formation of nitrogenous alkaline compounds [28,29]. The lower pH value on day 0 in MSBP might be mainly due to the addition of nisin and sodium lactate. Notably, the rate of pH increase in the MS group was lower than that in the CK group. Additionally, in the MSBP group, there was a significant decrease in the initial pH value by more than 0.2 (Figure 1A). After storing the crayfish tails at room temperature for 3 days, the pH values in the MS and MSBP groups were still lower than the value on day 1 in the CK group.

TVB-N, an indicator of protein degradation, is a valuable tool for assessing the freshness of meat [30]. The changes in TVB-N levels in the crayfish tails during storage are shown in Figure 1B. Initially, the three groups had the same TVB-N content. However, after 1 day of storage, the TVB-N significantly increased in the CK group, while only a slight increase was observed in the MS group, and TVB-N in the MSBP group remained almost unchanged. After 2 days, the TVB-N content in the CK group reached 30 mg/100 g. In contrast, the TVB-N content in the MSBP group was only 13 mg/100 g after 3 days of storage at room temperature. These results indicate that the deterioration of crayfish tails was lower in the MS and MSBP groups than in the CK group.

### 3.3. Changes in Volatile Compounds during Room Temperature Storage of Precooked Crayfish Tails

**Figure 2:**
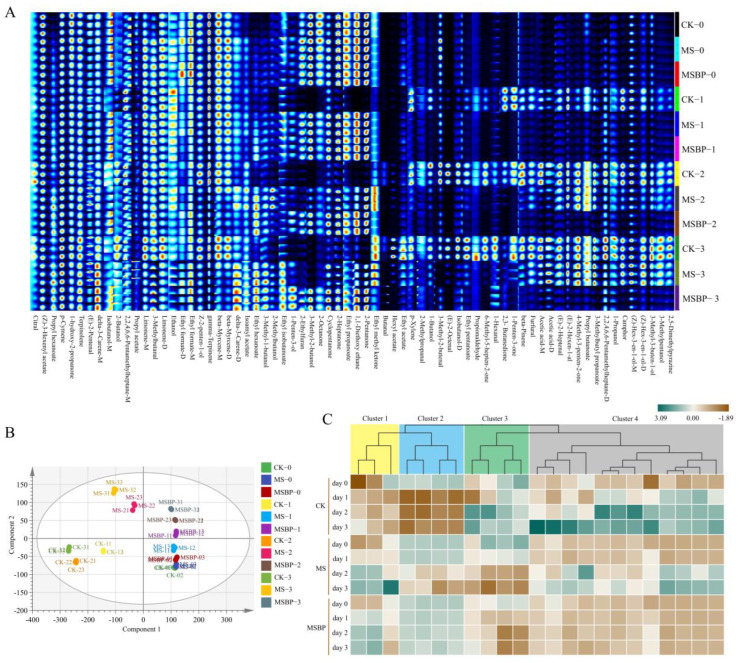
Fingerprint map of volatile compounds in precooked seasoned crayfish tails during storage at room temperature (**A**); OPLS-DA difference analysis of volatile compounds in precooked seasoned crayfish tails during storage at room temperature (**B**); and relative concentration cluster analysis of volatile compounds with VIP greater than 1 among different groups in precooked seasoned crayfish tails during storage at room temperature (**C**). The suffixes −0, 1, 2, and 3 represent storage days.

The flavor characteristics of food are strongly associated with the types and concentrations of volatile compounds [31]. In previous research, several volatile compounds were identified in cooked crayfish [19]. In this study, a total of 79 volatile compounds were detected in the precooked crayfish tails, of which 70 volatile compounds were identified (Table S1). These 70 compounds belonged to various chemical families, including 15 alcohols, 14 esters, 13 aldehydes, 11 ketones, nine terpenes, two acids, two aromatic hydrocarbons, two alkanes, one pyrazine, and one furan. To compare the volatile compounds among different groups, fingerprinting analysis was performed (Figure 2A). On day 0, the volatile compounds in the CK, MS, and MSBP groups were similar, with approximately half of the identified volatile compounds being detected. After storage at room temperature for 1 day, more than 30 new volatile compounds were produced in the CK group. However, the levels of the compounds 2-pentanone, 1,1-diethoxy ethane, ethyl propanoate, 2-heptanone, cyclopentanone, 2-octanone, 3-methyl-2-butanol, and 2-ethylfuran in the CK group decreased or disappeared as the storage time increased. In comparison, the volatile compounds in the MS group began to change after 2 days of room temperature storage. Remarkably, the volatile compounds in the MSBP group remained relatively unchanged, even after 3 days of storage at room temperature.

During the storage of aquatic products, the growth of microorganisms is a primary contributor to spoilage [32,33]. Metabolites such as aldehydes, ketones, and esters, produced by spoilage microorganisms, have significant effects on the odor of the products [17,34]. OPLS-DA was used to discriminate the crayfish tails using the relative concentration of volatile compounds (Figure 2B). The model exhibited good discrimination performance, as indicated by the R^2^X_cum_ value of 0.997, R^2^Y_cum_ value of 0.86, and Q^2^_cum_ value of 0.818. To ensure the reliability of the model and exclude overfitting, permutation tests were conducted for 200 iterations, resulting in R^2^ and Q^2^ intercepts of 0.151 and −0.49, respectively (Figure S1). These results further validate the robustness of the model. The OPLS-DA score plot clearly showed similar volatile compounds on day 0 among CK, MS, and MSBP. After storing at room temperature for 1 day, the volatile compounds in CK showed an obvious separation from MS and MSBP. The separation in MS began from day 2. As the storage time increased, the volatile compounds in MSBP were almost unchanged. The change in volatile compounds is consistent with the microorganisms in the precooked crayfish tails during room temperature storage.

Variable importance factor (VIP) scores are commonly used to determine the contribution of each compound in the PLS model. Compounds with a VIP score greater than one are considered essential for discrimination [35]. In our OPLS-DA model, we identified 23 volatile compounds with VIP scores greater than one (Table S1). A heatmap was constructed to analyze the changes in the concentrations of these compounds during storage (Figure 2C). Based on the clustering of the compounds, the compounds could be categorized into four groups, consisting of 3, 4, 4, and 12 compounds, respectively. Overall, the concentrations of volatile compounds in clusters 1 and 4 increased during storage, while the concentrations of the volatile compounds in clusters 2 and 3 decreased compared with those on day 0. We hypothesized that the volatile compounds in clusters 1 and 4 might be produced by microbial growth, whereas the compounds in clusters 2 and 3 most likely originated from the soup, seasoned oil, and boiled crayfish tails.

In cluster 1, the concentration of volatile compounds remained relatively stable in the CK group during storage but significantly increased in the groups treated with MS and MSBP. The main volatile compounds in cluster 1 were esters, such as propyl hexanoate and ethyl hexanoate. Esters play important roles in the flavor of fermented fishery products [36]. Ethyl hexanoate contributes to the sweet and fruity aroma of fermented common carp and sausage [37,38]. More than half of the essential volatile compounds were categorized into cluster 4. The concentrations of these compounds showed an opposite trend compared with those in cluster 1. In the control group, the concentrations of these compounds sharply increased beginning on day 1, while in the MS group, they gradually increased beginning on day 2 and remained unchanged in the MSBP group. These results indicate that microwave sterilization and biological preservatives promote the production of volatile compounds in cluster 1 but inhibit the production of volatile compounds in cluster 4. In meat products, most of the volatile compounds produced during storage are attributed to spoilage microorganisms [39,40]. The changes observed in volatile compounds in cluster 4 reflect the growth of spoilage microorganisms in the control group, which is consistent with the higher TVC observed. Therefore, the volatile compounds in cluster 4 are likely spoilage volatile compounds produced by spoilage microorganisms. In cluster 2, the concentrations of the compounds sharply decreased from day 1 in the control group and started to decrease from day 2 in the MS group but remained unchanged in the MSBP group even after 3 days of storage. The compounds in this cluster include 2-pentanone, 1,1-diethoxy ethane, 3-methy-2-butanol, and 2-heptanone. Ketones can be produced through the thermal oxidative degradation of unsaturated fatty acids or amino acids [41]. 2-Pentanone and 2-heptanone are found in fermented food and contribute to the unique flavors of meat products, such as floral, fruity, and cheesy flavors [38,42,43]. In this study, the sterilization and inhibition of microorganisms in the MSBP group reduced the changes in 2-pentanone and 2-heptanone during the storage of precooked crayfish tails, thus preserving the special flavor from the soup, seasoned oil, and boiled crayfish tails. Overall, these results demonstrate that the MSBP treatment effectively inhibits the deterioration of precooked crayfish tails during storage at room temperature.

### 3.4. Changes in Microbial Community during Room Temperature Storage of Precooked Crayfish Tails

**Figure 3:**
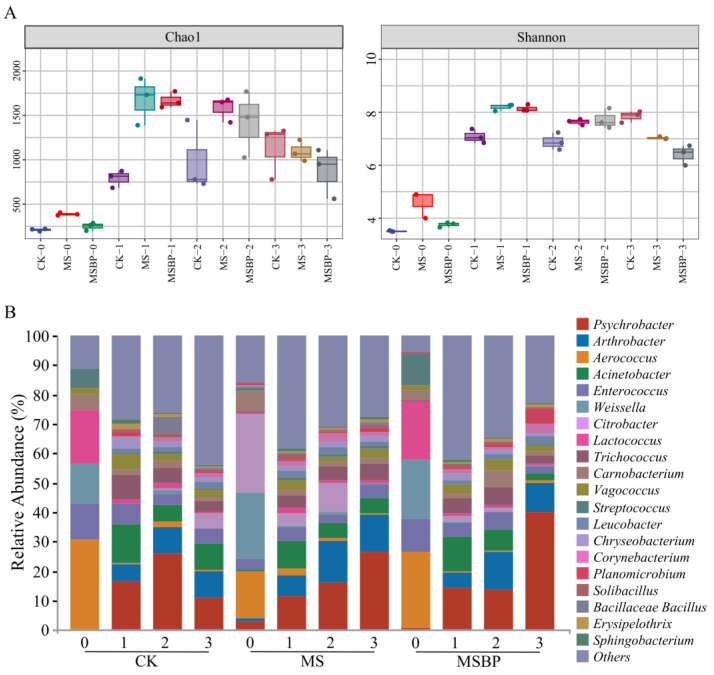
Alpha diversity analyzed using Chao1 and Shannon indices (**A**) and microbial communities and succession at the genus level (**B**) of the bacteria in precooked seasoned crayfish tails during storage at room temperature. The dots in (**A**) represent triplicate values.

The microbial composition of the samples was determined using 16S RNA sequencing. The alpha diversity, which indicates the richness and diversity of the microbial communities, was analyzed [44]. Figure 3A shows the Chao1 and Shannon index values, which are measures of richness and diversity, respectively. The values were initially lower on day 0, suggesting lower richness and diversity. However, after storage at room temperature, the richness and diversity increased across all the groups. In the CK group, the richness and diversity were similar on days 1 and 2 but increased on day 3. On the other hand, in the samples treated with MS and MSBP, the microbial communities showed the highest richness and diversity on day 1 and gradually decreased after day 2. To further explore the changes and similarities in community composition, clustering analysis was performed based on the top 10 bacterial genera (Figure S2). The results indicated that the bacterial compositions in the CK, MS, and MSBP groups on day 0 clustered closely together and differed from the post-storage compositions. Moreover, the bacterial compositions on day 1 and day 2 were clustered together, except for the CK group on day 2. Finally, the bacterial compositions in the MS and MSBP groups on day 3 were clustered together.

Figure 3B displays the top 20 bacterial genera with relatively higher relative abundances in precooked crayfish tails during storage. Initially, on day 0, *Aerococcus* had the highest proportion in the CK and MSBP groups, followed by *Lactococcus*, *Weissella*, *Enterococcus*, *Streptococcus*, and *Carnobacterium*. In the MS group, *Citrobacter* had the highest proportion on day 0, followed by *Weissella*, *Aerococcus*, *Carnobacterium*, and *Enterococcus*. It is worth noting that *Lactococcus*, *Weissella*, *Enterococcus*, and *Carnobacterium* are known to be spoilage microorganisms in food [45]. During the storage of precooked crayfish tails, there were significant changes in the bacterial communities. After storage at room temperature for more than one day, the dominant bacteria in all groups shifted to *Psychrobacter*, followed by *Acinetobacter* or *Arthrobacter*. Previous studies have identified *Psychrobacter* and *Acinetobacter* as the dominant bacteria in cooked crayfish muscle [46]. *Acinetobacter* was observed to grow better at room temperature than at lower temperatures (4 °C or 1 °C) during the storage of *Litopenaeus vannamei* [47]. This observation could explain the greater proportion of *Acinetobacter* in crayfish stored at room temperature. In the CK group, the abundance of *Psychrobacter* increased from 16.57% to 25.79% on day 2 but decreased to 10.89% on day 3. The abundance of *Acinetobacter* decreased on day 2 and increased on day 3. In the MS group, the abundances of *Psychrobacter* and *Arthrobacter* gradually increased from 11.56% to 26.51% and from 6.97% to 12.30%, respectively, as the storage time increased. In the MSBP group, the abundance of *Arthrobacter* increased to 12.71% on day 2 compared with that on day 1 (5.20%), while the abundance of *Acinetobacter* decreased from 11.58% to 7.08%. The abundance of *Psychrobacter* in MSBP increased to 40.07% on day 3. It is worth noting that *Psychrobacter* dominates in fishery products stored in air compared with those stored in vacuum and modified atmosphere packaging (75% CO_2_ and 25% N_2_) because of its preference for aerobic conditions [48,49]. In our study, the precooked crayfish tails were packaged with 5% O_2_, which favored the growth of *Psychrobacter*. However, because of the high growth rate in the CK group, limited oxygen inhibited the growth of *Psychrobacter*, resulting in a reduction in growth on day 3. Furthermore, it has been documented that other spoilage microorganisms can easily inhibit species of *Psychrobacter* [50]. These findings reveal the dynamic changes in the composition of spoilage microorganisms during the storage of precooked crayfish tails at room temperature and emphasize the influence of microwave sterilization and biological preservatives on microbial populations.

### 3.5. Correlation of Microorganisms and Flavor Compounds during Room Temperature Storage of Precooked Crayfish Tails

**Figure 4:**
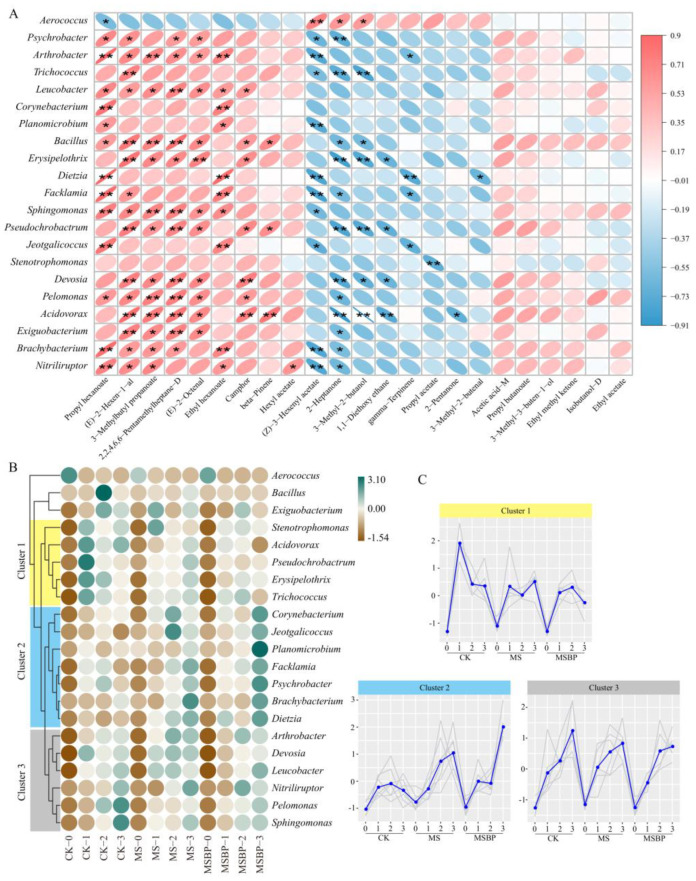
Correlation analysis between microbial communities and volatile compounds with a VIP greater than 1 in precooked seasoned crayfish tails during storage at room temperature (**A**); relative abundance cluster analysis of correlated bacteria (**B**); and abundance tendencies of the clustered bacteria (**C**). Asterisks stand for significant differences, *: *p* < 0.05, **: *p* < 0.01. The gray lines in subfigure (**C**) represent the actual abundances of the bacteria in the cluster.

During the storage of fishery products, the presence of volatile compounds is primarily due to the decomposition of fish constituents and the activity of spoilage microorganisms [48,51]. To understand the relationships between the microbial community and volatile compounds, we employed the Spearman algorithm to analyze the correlations. The results showed a significant correlation (*p* < 0.05, *r* > 0.7) between the microbes and volatile compounds (Figure 4A). Specifically, 21 bacteria were found to be significantly correlated with 17 volatile compounds (*p* < 0.05). The heatmap displays the relative abundances of these bacteria in the different storage groups (Figure 4B). Upon careful examination, it was observed that most of these bacteria had a relatively low abundance on day 0 in all three groups, but their abundance significantly increased after storage at room temperature for more than 1 day. Among the correlated bacteria, *Aerococcus* exhibited a different correlation pattern with volatile compounds compared with the other bacteria. While it showed a negative correlation with propyl hexanoate (*p* < 0.05), it exhibited a significant positive correlation with (Z)-3-hexenyl acetate, 2-heptanone, and 3-methyl-2-butanol. Additionally, half of the correlated volatile compounds, including propyl hexanoate, (E)-2-hexen-1-al, 3-methylbutyl propanoate, 2,2,4,6,6-pentamethylheptane-D, (E)-2-octenal, ethyl hexanoate, camphor, β-pinene, and hexyl acetate, were positively correlated with most of the bacteria. These correlated bacteria can be divided into the following two main groups: *Leucobacter*, *Bacillus*, *Erysipelothrix*, *Pseudochrobactrum*, *Devosia*, *Pelomonas*, and *Acidovorax*. The relative abundances of these bacteria displayed a clustered pattern, with clusters 1 and 3 representing their growth dynamics during storage. The abundance of these bacteria was significantly lower on day 0 but increased sharply after storage for more than 1 day. Notably, compared with those in the CK group, the abundance of bacteria in cluster 1 and the growth rate of bacteria in cluster 3 were lower in the groups treated with MS and microwave sterilization combined with nisin and sodium lactate MSBP (Figure 4C). These bacteria exhibited a significant positive correlation with (E)-2-hexen-1-al, 3-methylbutyl propanoate, 2,2,4,6,6-pentamethylheptane-D, (E)-2-octenal, and camphor, which significantly increased in the CK group but remained unchanged in the MSBP group as the storage time increased (Figure 2C). These findings suggest that *Leucobacter*, *Erysipelothrix*, *Pseudochrobactrum*, *Devosia*, *Pelomonas*, and *Acidovorax* may be the dominant spoilage microorganisms contributing to the production of volatile compounds in precooked crayfish tails during storage. The combination of microwave sterilization with nisin and sodium lactate partially inhibited the growth of spoilage microorganisms in precooked crayfish tails during storage at room temperature. Other bacteria, including *Corynebacterium*, *Planomicrobium*, *Dietzia*, *Facklamia*, and *Jeotgalicoccus*, showed a positive correlation with propyl hexanoate and ethyl hexanoate. As mentioned earlier, propyl hexanoate and ethyl hexanoate contribute to the unique flavor of fermented fish. Hence, it can be inferred that the bacteria in this group may contribute to the fermentation process of crayfish tails and enhance their flavor. Overall, the analysis of the microbial community and volatile compounds provides valuable insights into the spoilage and fermentation processes of fishery products during storage.

Contrary to the findings mentioned above, some volatile compounds, including (Z)-3-hexenyl acetate, 2-heptanone, 3-methyl-2-butanol, 1,1-diethoxy ethane, γ-terpinene, propyl acetate, 2-pentanone, and 3-methyl-2-butenal, exhibited a negative correlation with the majority of the bacteria (Figure 4A). Specifically, (Z)-3-hexenyl acetate had a negative association with *Psychrobacter*, *Arthrobacter*, *Trichococcus*, *Planomicrobium*, *Dietzia*, *Facklamia*, *Sphingomonas*, *Jeotgalicoccus*, *Brachybacterium*, and *Nitriliruptor*. Similarly, 2-heptanone was negatively correlated with *Psychrobacter*, *Trichococcus*, *Bacillus*, *Erysipelothrix*, *Facklamia*, *Pseudochrobactrum*, *Devosia*, *Pelomonas*, *Acidovorax*, *Exiguobacterium*, *Brachybacterium*, and *Nitriliruptor*. Additionally, 3-methyl-2-butanol and 1,1-diethoxy ethane were negatively associated with *Erysipelothrix*, *Pseudochrobactrum*, *Devosia*, and *Acidovorax*. γ-Terpinene had a negative correlation with *Arthrobacter*, *Dietzia*, *Facklamia*, and *Jeotgalicoccus*. Finally, propyl acetate, 2-pentanone, and 3-methyl-2-butenal were found to be negatively correlated with *Stenotrophomonas*, *Acidovorax*, and *Dietzia*, respectively. Most of these volatile compounds initially had higher concentrations on day 0 but decreased as the storage time increased, particularly in the MS and MSBP samples (Figure 2C, clusters 2 and 3). This suggests that these volatile compounds were potentially produced by the seasoned oil and soup. However, as the storage time increased, the concentrations of these volatile compounds decreased because of the production of other volatile compounds by the spoilage microorganisms. Interestingly, most bacteria exhibited a dual correlation, both negative and positive, with the related volatile compounds. These results imply that spoilage microorganisms may impact the volatile compounds in crayfish tails during storage in two distinct ways. First, spoilage microorganisms contribute to the production of volatile compounds associated with spoilage through microbial activities. Second, the growth of spoilage microorganisms leads to a decrease in the flavor of volatile compounds generated during processing.

### 3.6. Predicted Functional Analysis of the Microbial Communities

**Figure 5:**
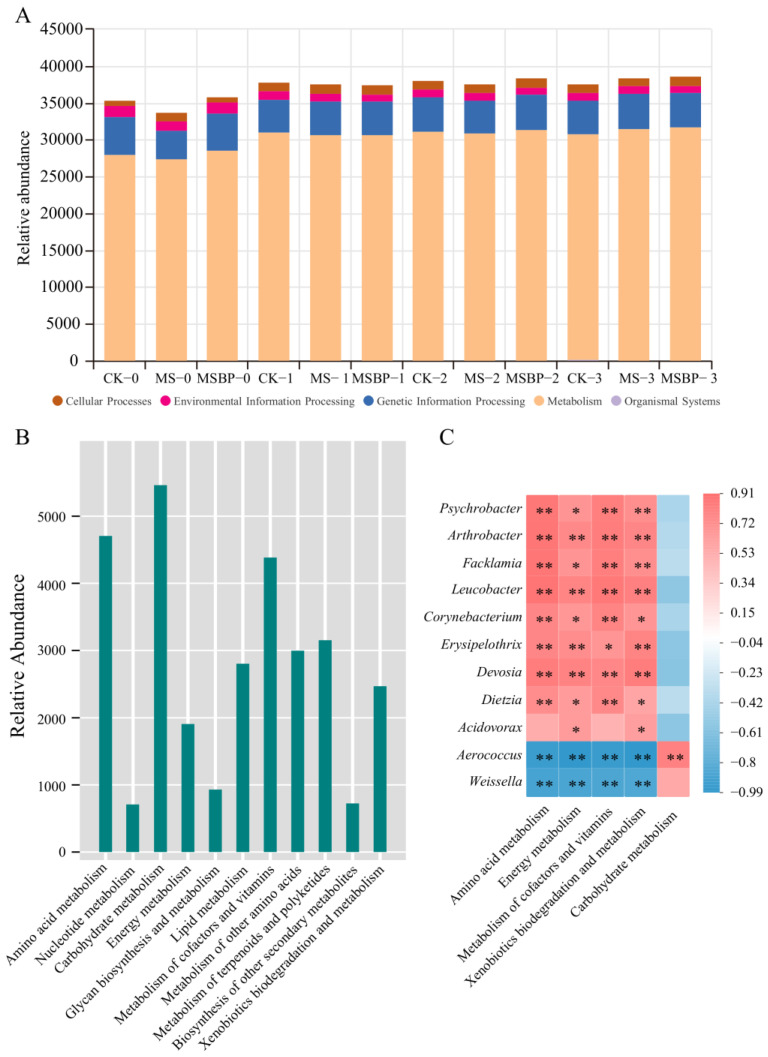
Predicted functions in KEGG pathways of the bacteria identified in precooked seasoned crayfish tails during storage at room temperature (**A**); relative abundance of metabolic pathways of the bacteria identified in precooked seasoned crayfish tail during storage at room temperature (**B**); and correlation analysis between microbial communities and altered metabolic pathways in precooked seasoned crayfish tails during storage at room temperature (**C**). Asterisks stand for significant differences, *: *p* < 0.05, **: *p* < 0.01.

Microbial activity plays a crucial role in the spoilage of fish during storage [52]. The spoilage potential and activity of bacteria responsible for spoilage are closely related to their metabolic and enzymatic activities [53]. To gain a better understanding of the microbiota functions in crayfish tails during storage, we studied the predicted KEGG pathways associated with the microbiota using PICRUSt 2 [25]. Analysis of the results in Figure 5A revealed that the bacteria in the precooked crayfish tails stored at room temperature were primarily engaged in metabolic pathways, followed by genetic information processing, cellular processes, environmental information processing, and organismal systems. The predicted relative abundance of metabolism on day 0 in all three groups was lower than 30,000. After storage at room temperature for more than 1 day, the relative abundance of metabolism increased to more than 30,000 in all groups. We further analyzed the main metabolic pathways among the different groups during storage. The annotated metabolic pathways with abundant representation were carbohydrate metabolism, amino acid metabolism, metabolism of cofactors and vitamins, metabolism of other amino acids, metabolism of terpenoids and polyketides, xenobiotics biodegradation and metabolism, lipid metabolism, energy metabolism, and glycan biosynthesis and metabolism (Figure 5B). Among these pathways, amino acid metabolism, carbohydrate metabolism, energy metabolism, metabolism of cofactors and vitamins, and xenobiotics biodegradation and metabolism exhibited significant changes in the different groups during storage (Figure S3). After 1 day of storage, the relative abundance of carbohydrate metabolism decreased, while the abundance of amino acid metabolism, energy metabolism, metabolism of cofactors and vitamins, and xenobiotics biodegradation and metabolism increased. Crayfish are well-known for their high protein content and amino acid content in muscle, while the carbohydrate content is relatively low in crayfish tails [54]. This low carbohydrate and high protein composition inhibits the growth of bacteria with high carbohydrate metabolic activity while promoting the growth of bacteria with high amino acid metabolic activity. To evaluate the contributions of volatile compound-producing bacteria to metabolic activity, we performed a correlation analysis between the bacteria and the altered metabolic pathways (Figure 5C). Eleven bacteria were found to be correlated with the five metabolic pathways. *Psychrobacter*, *Arthrobacter*, *Facklamia*, *Leucobacter*, *Corynebacterium*, *Erysipelothrix*, *Devosia*, and *Dietzia* exhibited positive correlations with amino acid metabolism, energy metabolism, metabolism of cofactors and vitamins, and xenobiotics biodegradation and metabolism pathways. Conversely, *Aerococcus* and *Weissella* exhibited negative correlations with these pathways. *Acidovorax* was positively correlated with energy metabolism and xenobiotics biodegradation and metabolism pathways. Carbohydrate metabolism was only positively correlated with *Aerococcus*. These findings suggest that changes in volatile compounds in precooked crayfish tails during storage at room temperature may be attributed to amino acid metabolism, energy metabolism, metabolism of cofactors and vitamins, and xenobiotics biodegradation and metabolism pathways of dominant bacteria such as *Psychrobacter*, *Arthrobacter*, *Facklamia*, *Leucobacter*, *Corynebacterium*, *Erysipelothrix*, *Devosia*, *Dietzia*, and *Acidovorax*.

## 4. Conclusions

Our previous study investigated the effects of the appropriate microwave time and concentration of sodium lactate on the microorganisms and qualities of seasoned crayfish tails stored at room temperature and prolonged the shelf life of precooked crayfish tails at room temperature for up to 3 days. In the present study, we demonstrated that microwave treatment combined with sodium lactate and the antimicrobial peptide nisin had a noticeable impact on the spoilage of prefabricated crayfish tails stored at room temperature with TVC, pH, TVB-N, and volatile compounds used as the evaluation indices. The volatile compounds detected during storage were closely associated with the presence of specific bacteria. Notably, *Psychrobacter*, *Arthrobacter*, *Facklamia*, *Leucobacter*, *Corynebacterium*, *Erysipelothrix*, *Devosia*, *Dietzia*, and *Acidovorax* were identified as the dominant bacteria that potentially contributed to the changes in volatile compounds. Their activities in amino acid metabolism, energy metabolism, cofactor and vitamin metabolism, and xenobiotics biodegradation and metabolism likely played a significant role in shaping the volatile profiles during storage.

This comprehensive study provides important insights into the changes in volatile compounds, microbial communities, and their interrelationships in precooked seasoned crayfish tails stored at room temperature. The findings lay the groundwork for further strategies aimed at preventing spoilage in this product under room temperature storage conditions.

## Figures and Tables

**Table 1:** Total TVC in precooked seasoned crayfish tail during storage at room temperature.

Groups	Total Viable Count (log CFU/g)			
	Day 0	Day 1	Day 2	Day 3
CK	4.82 ± 0.11 ^a^	7.98 ± 0.01 ^a^	9.52 ± 0.06 ^a^	10.81 ± 0.02 ^a^
MS	1.43 ± 0.60 ^b^	2.40 ± 0.02 ^b^	6.62 ± 0.08 ^b^	7.82 ± 0.06 ^b^
MSBP	1.15 ± 0.21 ^b^	2.04 ± 0.05 ^b^	2.67 ± 0.08 ^c^	4.15 ± 0.01 ^c^

Notes: Values are expressed as the mean ± standard deviation. Different letters in the column indicate that there are significant differences between groups.

## Data Availability

The original contributions presented in this study are included in this article/Supplementary Materials. Further inquiries can be directed to the corresponding authors.

## Supplementary Materials

The following supporting information can be downloaded at: https://www.mdpi.com/article/10.3390/foods13081256/s1, Table S1: Volatile compounds identified in different periods of precooked seasoned crayfish tails during storage at room temperature. Figure S1: Permutation test of the OPLS-DA model. Figure S2: Hierarchical clustering analysis of the bacteria in precooked seasoned crayfish tails during storage at room temperature. Figure S3: Relative abundance of KEGG metabolic pathways in precooked seasoned crayfish tails with different treatments during storage at room temperature.
