# Supplementary material for: Changes in the Quality and Microbial Communities of Precooked Seasoned Crayfish Tail Treated with Microwave and Biological Preservatives during Room Temperature Storage
Source: Foods. 2024 Apr 19;13(8):1256. doi: 10.3390/foods13081256 (PMC11049464; doi:10.3390/foods13081256)
Supplement: Supplementary file 1 [file foods-13-01256-s001.zip › foods-2934913-supplementary.pdf]

Table S1. Volatile compounds identified in different periods of pre-cooked seasoned crayfish tail during storage at room temperature.

Figure S1. Permutation test of OPLS-DA model.

Figure S2. Hierarchical clustering analysis of the bacteria in pre-cooked seasoned crayfish tail during storage at room temperature.

Figure S3. Relative abundance of KEGG metabolic pathways in pre-cooked seasoned crayfish tail with different treatments during storage at room temperature.

Table S1: Volatile compounds identified in different periods of pre-cooked seasoned crayfish tail during storage at room temperature.

	Compounds	CAS#	Formula	MW	RI	Rt [sec]	VIP
1	Citral	5392405	$C_{10}H_{16}O$	152.2	1711.4	1324.688	0.803546
2	Camphor	76222	$C_{10}H_{16}O$	152.2	1511.2	983.02	1.38035
3	Acetic acid-M	64197	$C_2H_4O_2$	60.1	1397.9	830.256	1.61889
4	Acetic acid-D	64197	$C_2H_4O_2$	60.1	1394.9	825.328	0.792033
5	(E)-2-Octenal	2548870	$C_8H_{14}O$	126.2	1396.7	828.613	1.56695
6	(Z)-Hex-3-en-1-ol-M	928961	$C_6H_{12}O$	100.2	1362.9	767.889	0.866588
7	(Z)-Hex-3-en-1-ol-D	928961	$C_6H_{12}O$	100.2	1362.8	767.708	0.399673
8	6-Methyl-5-hepten-2-one	110930	$C_8H_{14}O$	126.2	1347.6	742.044	0.623641
9	(E)-2-Heptenal	18829555	$C_7H_{12}O$	112.2	1318.7	695.179	0.987433
10	(Z)-3-Hexenyl acetate	3681718	$C_8H_{14}O_2$	142.2	1312.6	685.695	1.19942
11	Propyl hexanoate	626777	$C_9H_{18}O_2$	158.2	1316.9	692.39	1.05472
12	2,5-Dimethylpyrazine	123320	$C_6H_8N_2$	108.1	1318.3	694.621	0.763298
13	p-Cymene	99876	$C_{10}H_{14}$	134.2	1312.6	685.695	0.856795
14	1-Hydroxy-2-propanone	116096	$C_3H_6O_2$	74.1	1304.9	673.978	0.530654
15	Furfural	98011	$C_5H_4O_2$	96.1	1445.3	891.006	0.606791
16	Terpinolene	586629	$C_{10}H_{16}$	136.2	1272.7	614.84	0.534729
17	Z-2-penten-1-ol	1576950	$C_5H_{10}O$	86.1	1270.7	610.934	0.590488
18	3-Methyl-3-buten-1-ol	763326	$C_5H_{10}O$	86.1	1253.7	578.575	1.26208
19	3-Methyl-2-butenal	107868	C_5H_8O	84.1	1223.1	524.707	1.03886
20	gamma-Terpinene	99854	$C_{10}H_{16}$	136.2	1229.3	535.301	1.6975
21	Limonene-M	138863	$C_{10}H_{16}$	136.2	1229.5	535.667	0.799781
22	Limonene-D	138863	$C_{10}H_{16}$	136.2	1229.3	535.301	0.614356
23	(E)-2-Hexen-1-al	6728263	$C_6H_{10}O$	98.1	1200.2	487.809	1.33343
24	3-Methylbutyl propanoate	105680	$C_8H_{16}O_2$	144.2	1198.3	484.881	1.46689
25	p-Xylene	106423	C_8H_{10}	106.2	1154.4	425.412	0.839299
26	4-Methyl-3-penten-2-one	141797	$C_6H_{10}O$	98.1	1147.8	417.194	0.891425
27	(E)-2-Pentenal	1576870	C_5H_8O	84.1	1147.9	417.355	0.931108
28	delta-3-Carene-M	13466789	$C_{10}H_{16}$	136.2	1133.8	400.434	0.8297
29	delta-3-Carene-D	13466789	$C_{10}H_{16}$	136.2	1134.7	401.401	0.622977
30	1-Butanol	71363	$C_4H_{10}O$	74.1	1124.4	389.475	0.820509
31	Isoamyl acetate	123922	$C_7H_{14}O_2$	130.2	1125.3	390.442	0.315939
32	Isobutanol-D	78831	$C_4H_{10}O$	74.1	1097.3	359.662	1.00447
33	Isobutanol-M	78831	$C_4H_{10}O$	74.1	1092.1	356.323	0.676137
34	beta-Pinene	127913	$C_{10}H_{16}$	136.2	1095.3	358.373	1.1295
35	1-Hexanal	66251	$C_6H_{12}O$	100.2	1088.2	353.861	0.522142
36	1-Propanol	71238	C_3H_8O	60.1	1036	322.218	0.861225
37	1-Penten-3-one	1629589	C_5H_8O	84.1	1014	309.702	0.493576
38	2,3-Butanedione	431038	$C_4H_6O_2$	86.1	1017.6	311.752	0.401
39	2-Butanol	78922	$C_4H_{10}O$	74.1	1014.2	309.839	0.812746
40	2-Pentanone	107879	$C_5H_{10}O$	86.1	1013.7	309.566	2.29757

41	2,2,4,6,6-Pentamethylheptane-M	13475826	$C_{12}H_{26}$	170.3	976.8	289.698	0.469456
42	2,2,4,6,6-Pentamethylheptane-D	13475826	$C_{12}H_{26}$	170.3	977.7	290.192	1.73526
43	3-Methylbutanal	590863	$C_5H_{10}O$	86.1	922	262.562	0.445584
44	Ethyl methyl ketone	78933	C_4H_8O	72.1	888.8	247.385	1.00608
45	Ethanol	64175	C_2H_6O	46.1	924.1	263.549	0.141227
46	Butanal	123728	C_4H_8O	72.1	860.1	234.933	0.587155
47	Ethyl acetate	141786	$C_4H_8O_2$	88.1	849.5	230.492	1.16914
48	Propionaldehyde	123386	C_3H_6O	58.1	755.2	194.589	0.453217
49	Ethyl formate-M	109944	$C_3H_6O_2$	74.1	794.7	208.9	0.299637
50	Ethyl formate-D	109944	$C_3H_6O_2$	74.1	796.2	209.462	0.404773
51	2-Methylpropanal	78842	C_4H_8O	72.1	759.6	196.164	0.380813
52	2-Heptanone	110430	$C_7H_{14}O$	114.2	1196.4	481.863	1.52378
53	Cyclopentanone	120923	C_5H_8O	84.1	1209.3	502.218	0.689867
54	2-Ethylfuran	3208160	C_6H_8O	96.1	966.9	284.597	0.81774
55	Ethyl propanoate	105373	$C_5H_{10}O_2$	102.1	969.3	285.833	0.834446
56	1,1-Diethoxy ethane	105577	$C_6H_{14}O_2$	118.2	886.4	246.283	2.37805
57	3-Methyl-2-butanol	598754	$C_5H_{12}O$	88.1	1098.8	361.226	1.66887
58	Propyl acetate	109604	$C_5H_{10}O_2$	102.1	1007	305.855	1.01831
59	2-Octanone	111137	$C_8H_{16}O$	128.2	1295.2	659.437	0.445775
60	2-Methylbutanol	137326	$C_5H_{12}O$	88.1	1198.2	484.629	0.71046
61	Ethyl hexanoate	123660	$C_8H_{16}O_2$	144.2	1230.2	536.824	1.47315
62	Propyl butanoate	105668	$C_7H_{14}O_2$	130.2	1147.8	417.275	1.51622
63	Ethyl pentanoate	539822	$C_7H_{14}O_2$	130.2	1113.8	377.504	0.525031
64	Hexyl acetate	142927	$C_8H_{16}O_2$	144.2	1253.6	578.397	1.09504
65	3-Methyl-1-butanol	123513	$C_5H_{12}O$	88.1	1244.4	561.705	0.670686
66	1-Penten-3-ol	616251	$C_5H_{10}O$	86.1	1207.8	499.752	0.464259
67	Ethyl isobutanoate	97621	$C_6H_{12}O_2$	116.2	918.2	260.758	0.180973
68	beta-Myrcene-M	123353	$C_{10}H_{16}$	136.2	1216.5	513.807	0.951909
69	beta-Myrcene-D	123353	$C_{10}H_{16}$	136.2	1216.7	514.112	0.70824
70	3-Methylpentanol	589355	$C_6H_{14}O$	102.2	1316.5	691.752	0.575732

Notes: RI, retention index; Rt, retention time in the capillary GC column; the suffixes -M represents monomer, the suffixes -D represents dimer.

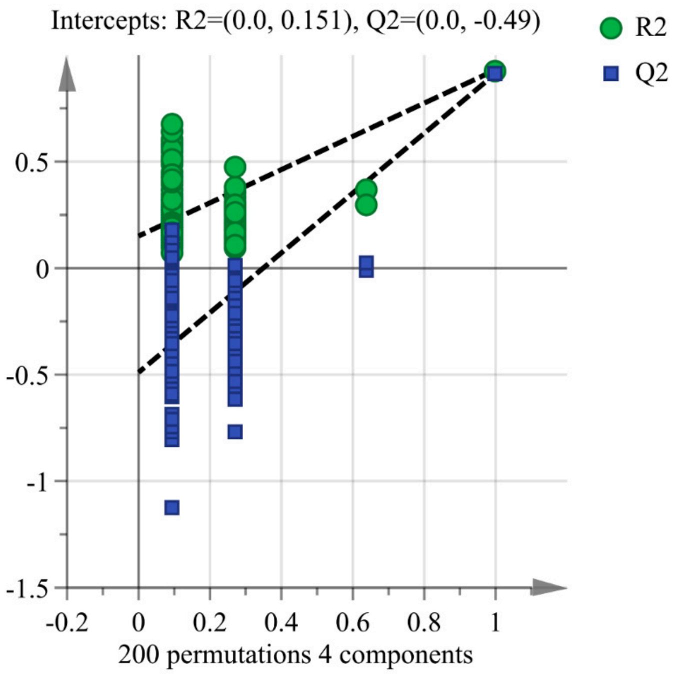

Figure S1. Permutation test of OPLS-DA model.

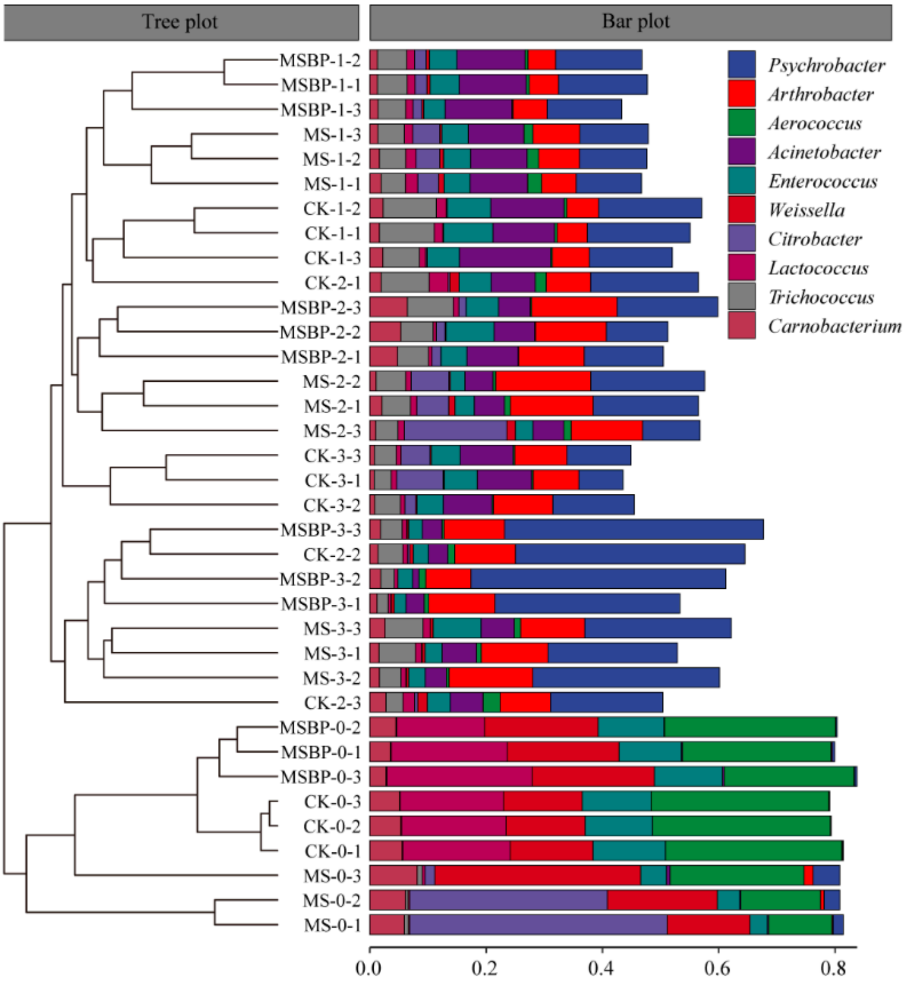

Figure S2. Hierarchical clustering analysis of the bacteria in pre-cooked seasoned crayfish tail during storage at room temperature.

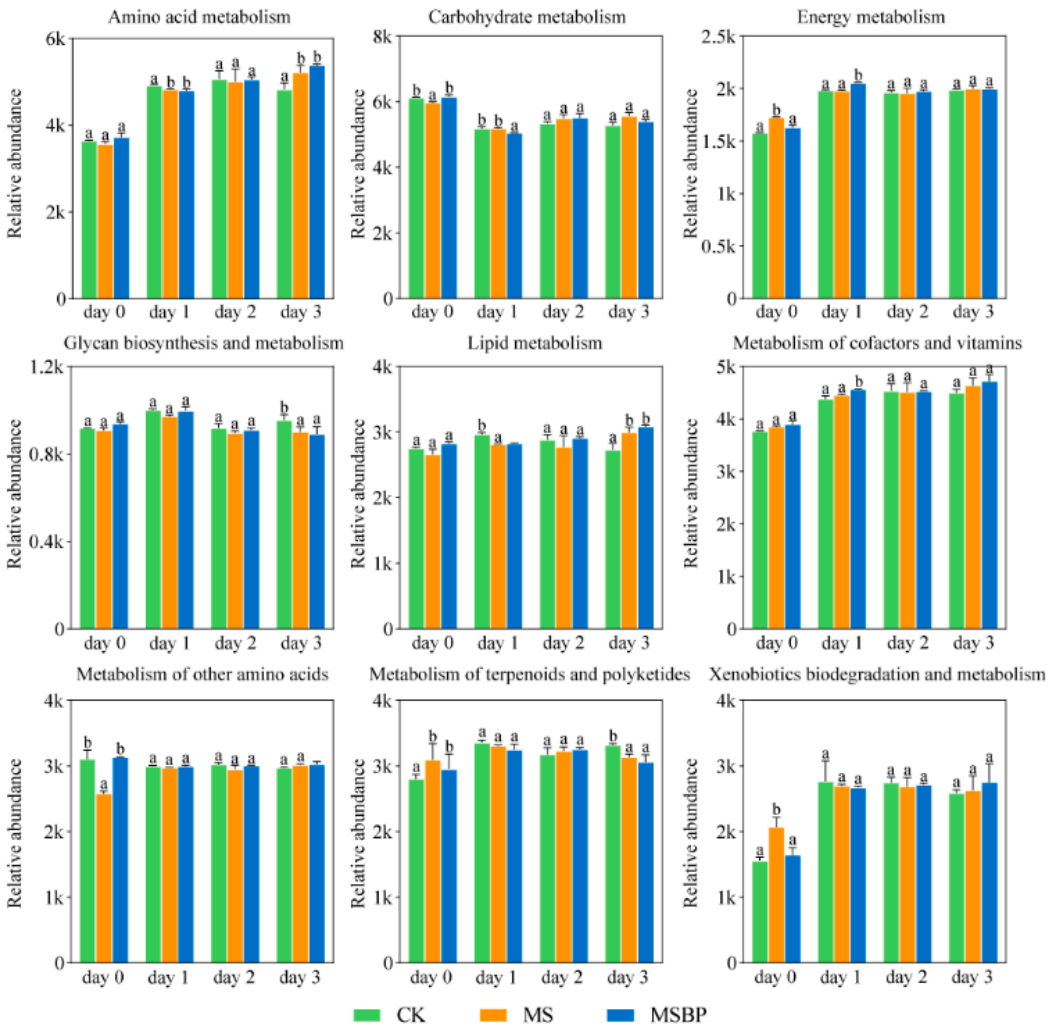

Figure S3. Relative abundance of KEGG metabolic pathways in pre-cooked seasoned crayfish tail with different treatments during storage at room temperature. Different letters in the figures indicate that there are significant differences between groups at the same day.
